# Education Research: Can Large Language Models Match MS Specialist Training? A Comparative Study of AI and Student Responses to Support Neurology Education

**DOI:** 10.1212/NE9.0000000000200260

**Published:** 2025-11-20

**Authors:** Hernan Inojosa, Ahmadreza Ramezanzadeh, Iva Gasparovic-Curtini, Isabella Wiest, Jakob Nikolas Kather, Stephen Gilbert, Tjalf Ziemssen

**Affiliations:** 1 Center of Clinical Neuroscience, Department of Neurology, University Hospital Carl Gustav Carus, Technical University of Dresden, Germany; and; 2 Else Kröner Fresenius Center for Digital Health, Technical University Dresden, Germany.

## Abstract

**Background and Objectives:**

Artificial intelligence (AI), particularly large language models (LLMs), is increasingly explored for clinical decision support and medical education. While general LLM proficiency on broad medical examinations has been demonstrated, their application of domain-specific knowledge in neurology remains underexplored. This study addresses that gap using multiple sclerosis (MS) as an exemplar, evaluating how LLM information access strategies affect accuracy in a specialized postgraduate curriculum and exploring possible roles of LLMs in neurology education.

**Methods:**

A comparative evaluation was conducted using 53 multiple-choice questions (MCQs) and 21 open-ended questions drawn from an MS curriculum used in a postgraduate MS program. As a reference, results from postgraduate students—primarily neurologists and neurology trainees—were used. Each question was answered by 3 LLMs: GPT-4o (general-purpose), MS RAG (retrieval-augmented accessing MS literature), and Prof. Valmed (CE-certified domain-specific, trained on medical data). All models operated in the zero-shot mode without previous exposure to the items. Questions were stratified based on students' performance. Accuracy was compared using χ^2^ tests.

**Results:**

Among LLMs, GPT-4o reached 81.1% accuracy, MS RAG 86.8%, and Prof. Valmed 91.3% while the reference students' cohort (n = 28) achieved a mean of 82% (SD 23%). Although overall differences were not statistically significant (χ^2^(2) = 2.165, *p* = 0.339, Cramer *V* = 0.119), performance varied by question type and difficulty. For MCQs with a single correct answer, domain-specific LLMs outperformed GPT-4o, although differences remained nonsignificant. By contrast, students showed stronger performance on single-wrong answer formats. Stratified by difficulty, students outperformed LLMs on “easy” questions while LLMs tended to achieve higher accuracy on “medium” and “hard” items. For open-ended questions, students reached 77.8% accuracy while GPT-4o, MS RAG, and Prof. Valmed scored 66.7%–85.0%.

**Discussion:**

These findings indicate that while LLMs can perform at levels broadly comparable to postgraduate students, these may be particularly useful on more difficult tasks, where their consistency may complement human reasoning in a neurology subspecialty curriculum. While results should be interpreted cautiously given the limited sample size, this study illustrates possible implications of LLMs in neurology education—for example, as AI tutors for complex topics, as support for formative assessments, or as targeted review resources. Further research should assess integration into educational workflows and decision support.

## Introduction

Artificial intelligence (AI), particularly large language models (LLMs), is increasingly explored for medical education and clinical decision support, enabling self-directed learning and context-specific feedback.^1-3^ These models have shown promise in replicating elements of expert reasoning and retrieving medical knowledge across neurologic diseases, including conditions such as multiple sclerosis (MS).^4,5^

LLMs such as ChatGPT (OpenAI, San Francisco, CA) have demonstrated high performance on standardized examinations such as the United States Medical Licensing Examination, with GPT-4 achieving accuracy rates up to 90%.^6-13^ However, performance varies depending on task complexity and alignment between training data and specific medical context and less on specific prompting methods or inclusion of media elements.^3,14^ In neurology, particularly in MS, effective knowledge application requires nuanced interpretation of evolving clinical guidelines, an area where domain-aligned models may offer improved support.

Recent advancements in specialized models trained or fine-tuned on curated medical literature underscore the potential of LLMs to provide precise and clinically relevant recommendations.^15^ Integrating retrieval-augmented generation (RAG) frameworks may provide insights into how access to up-to-date medical information can enhance the quality and applicability of responses.^3^ The recently developed model “Prof. Valmed” may highlight the value of domain-specific fine-tuning in improving response accuracy and relevance.^16^ Yet, their role in formal neurology and MS education remains relatively underexplored. Unlike other specialties with standardized board certifications, MS education lacks uniform assessment frameworks, complicating benchmarking. Postgraduate programs such as the MS Management Master at Dresden International University (DIU) and the Charcot MS Master (M.Sc.) represent efforts to standardize MS certification and offer curated curricula suitable for comparative evaluation.^17,18^

This study addresses a foundational gap in neurology education research: while LLM proficiency on medical examinations has been established, their performance in specialized clinical domains—such as MS—has not been systematically compared with that of expert learners. MS training involves application of evolving treatment guidelines and context-sensitive clinical reasoning, areas where LLMs may offer structured support if properly aligned.

By conducting a structured comparison of multiple LLM architectures against student performance across a standardized MS question set, we evaluate the knowledge application capabilities of these models. The models included a general-purpose LLM (GPT-4o), a RAG model with dynamic access to literature (MS RAG), and a domain-specific model trained on curated medical literature (Prof. Valmed). While educational outcomes were not directly assessed, our study aims to provide a basis for understanding how LLMs might support specialized training in MS and other complex neurologic conditions and inform future applications in neurology education. Such applications may include AI tutors that provide individualized (guideline-based) feedback on complex scenarios, tools for education aligned with postgraduate curricula, or supplementary resources to expand exposure to subspecialty content with evolving evidence.

## Methods

We performed a cross-sectional study using a standardized set of questions designed to reflect key aspects of MS diagnosis, treatment, and management based on the MS Management Master's Program at DIU and the Charcot MS Master (M.Sc.). Model-generated answers were produced on December 31, 2024, for GPT-4o and the RAG model, and between April 28 and April 30, 2025, for Prof. Valmed. The study adhered to best practices in AI evaluation in health care, including compliance with ethical standards for research involving nonhuman datasets.

### Large Language Models

The 3 distinct configurations of LLMs for evaluation included the following:

1. GPT-4o: the latest GPT version from OpenAI and well-known state-of-the-art model trained on diverse datasets. Although GPT-4o is not specifically fine-tuned for medical applications, its versatility allows it to perform well across various domains.^19^ In this study, GPT-4o was accessed using the official online platform of OpenAI under default settings.
2. RAG model (MS RAG): a customized retrieval-augmented generation model built using GPT-4o through the GPT builder interface from OpenAI.^20^ The model was specifically tailored on approximately 516 MB of curated MS-specific material, including lecture scripts, medical guidelines, and curated teaching materials from the MS Management Master's Program at DIU. These materials were partially developed by the same teaching staff who also authored the standardized question set used in this study. MS RAG uses dynamic search and retrieval mechanisms to enhance the contextual relevance and specificity of its responses. The model was accessed through its publicly available instance.^21^
3. Prof. Valmed: a specialized RAG-augmented knowledge retrieval system with a medical data architecture comprising approximately 2.5 million validated documents, including medical guidelines and content relevant to MS. Prof. Valmed is originally designed to assist health care professionals by generating clinically precise and guideline-concordant recommendations as a medical device with CE class 2b status. Prof. Valmed (version 2.0) was accessed through its proprietary interface.^16^ Prof. Valmed's training data are independent from the educational materials used in this study and were not influenced by the academic staff who developed the curriculum.

### Dataset

The dataset comprised 53 multiple-choice questions sourced from the curricula of module 4 (“Disease-Modifying MS Treatment”) in both the MS Management Master's Program at the DIU and the Charcot MS Master. The questions were designed to provide comprehensive evaluation on MS covering topics such as efficacy profiles, safety monitoring, sequencing strategies, and therapeutic indications. The question set included 2 formats:

1. Single-correct answer questions: questions with 1 correct option among multiple distractors (standard multiple-choice format).
2. Single-wrong answer questions: questions with 1 wrong option among multiple distractors (standard multiple-choice format).

Questions were reviewed and approved by a panel of 3 independent MS specialists from the DIU for content validity, clarity, and guideline alignment. Distractors in the answer formats were specifically designed to reflect common misconceptions, thereby challenging the models to distinguish correct answers from plausible but incorrect options. Each question had a predetermined correct answer based on expert-validated clinical guidelines, serving as the gold standard for evaluating model accuracy.

### Reference Group

To contextualize model performance, a group of 28 MS master's students nearing the completion of a postgraduate MS management program served as a reference for human-level performance. The students, divided equally between German-speaking and international cohorts, primarily consisted of medical neurologists and neurologists in training, with few additional participants from related fields (e.g., biology or pharmaceutical industry, among others). They completed the set of questions in a virtual environment in real-life examination conditions.

### Prompting Strategy

Responses were generated under uniform conditions to maintain comparability. The models were first tasked with selecting the correct option from predefined answer choices. This format included single-correct answer questions and single-wrong answer questions. To ensure uniformity, each question was presented in isolation, and the models generated responses based solely on their existing knowledge without previous exposure to the questions assessed, adhering to a zero-shot prompting approach. In this study, models are tasked with generating responses without additional in-context examples or domain-specific tuning beyond the training data.^3,22^ Owing to its current configuration, Prof. Valmed was not tested on 2 questions requiring image interpretation because its response generation is restricted to text-based inputs and outputs.

A subset of questions appropriate for free-text responses was presented in an open-ended format, where the question text was displayed without predefined answer options, requiring the model to generate a free-text response. This subset excluded questions that inherently relied on structured formats, such as those requiring the identification of a single incorrect statement or those designed to compare multiple predefined options. All other available questions were included. This dual-format strategy allowed a comprehensive evaluation of the models' ability to identify and generate correct answers across both structured and unstructured scenarios. Because open-ended responses were only generated by the models, student performance was not available for direct comparison in this format. Accuracy of open-ended responses was qualitatively assessed based on previously predefined criteria.^23^ To determine whether the response appropriately addressed the clinical question posed. Each response was categorized relative to the gold standard as accurate, inaccurate, or indeterminate. Indeterminate responses were those that were vague or generic, only partially addressed the question, or lacked sufficient detail to determine clinical soundness. Two clinicians reviewed and categorized each response independently and were blinded to each other's answers (HI and TZ).

### Standard Protocol Approvals, Registrations, and Participant Consents

This study did not constitute human subjects research and did not require institutional review board approval. The need for informed consent was waived accordingly, consistent with ethical research standards studies using nonidentifiable data. Only anonymized, aggregate results were analyzed; no personal or sensitive information was collected, and no individual student performance data were assessed or reported.

### Statistical Analysis

All data were summarized and presented in descriptive tables and/or visualized using bar graphs and line graphs. Quantitative results for accuracy and comparative performance metrics were displayed as mean values with SDs, medians with interquartile ranges (IQRs), or percentages, as appropriate. Questions were stratified by difficulty based on student performance: easy (>80% answered correctly), medium (50%–79%), and hard (<50%). Model performance was subsequently analyzed within these difficulty categories to evaluate whether accuracy differed across question types. Statistical comparisons between models for accuracy across question difficulties and question types were conducted using χ^2^ tests, with a significance threshold set at *p* < 0.05. Accuracy was defined as the proportion of correct answers compared with the gold standard. For open-ended questions, accuracy outcomes were reported as proportions for each response category (accurate, inaccurate, indeterminate). All statistical analyses were performed using IBM SPSS Statistics (version 30.0.0.0; IBM Corp., Armonk, NY), and visualizations were created with GraphPad Prism (version 5; GraphPad Software, San Diego, CA).

### Data Availability

The datasets generated and analyzed during this study are available from the corresponding author on reasonable request. However, the multiple-choice and open-ended questions derived from the MS master's program curriculum are subject to academic use restrictions and cannot be publicly shared.

## Results

### Comparative Accuracy of Students and Models

**Figure 1:**
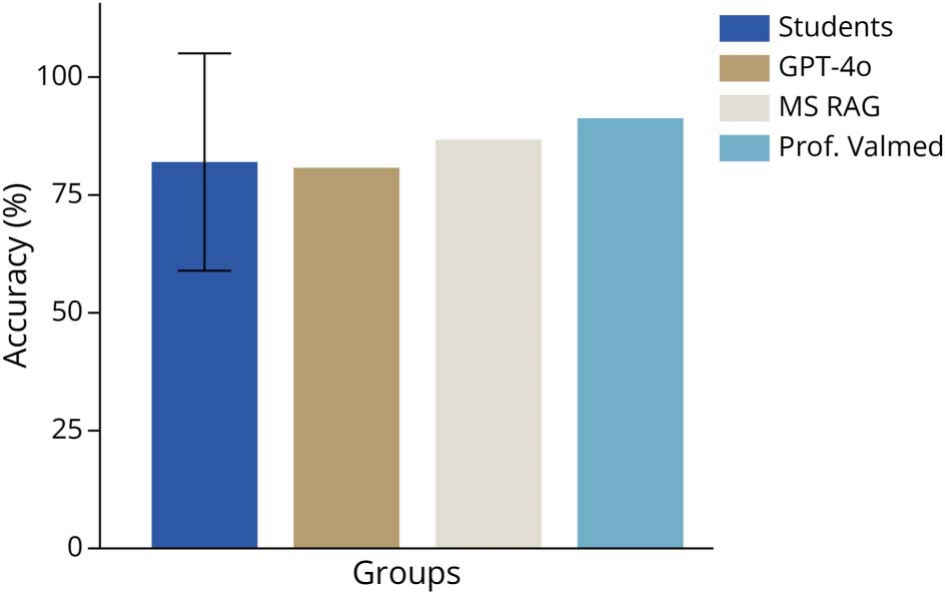
Overall Accuracy Across Groups Overall accuracy achieved by MS master's students (mean, SD) and 3 large language models (GPT-4o, MS RAG, and Prof. Valmed) in response to n = 53 multiple-choice questions. Prof. Valmed and MS RAG had a tendency toward higher accuracies, overall. However, no significant differences were observed in χ^2^ tests. MS = multiple sclerosis; RAG = retrieval-augmented generation.

A total of 53 questions were included in the study. The mean accuracy of the student cohort was 82.2% (SD 23%, median 92.8%, IQR 71%–100%, Figure 1). Among the models, GPT-4o achieved an accuracy of 81.1% and MS RAG achieved 86.8% while Prof. Valmed achieved 91.3%, calculated based on the questions it answered. No statistical significant difference was observed between the models (χ^2^(2) = 2.165, *p* = 0.339) with a small effect size (Cramer *V* = 0.084). Seven questions were left unanswered by Prof. Valmed (referral to a safety mechanism) and excluded from accuracy computation.

### Performance by Multiple-Choice Question Type

**Figure 2:**
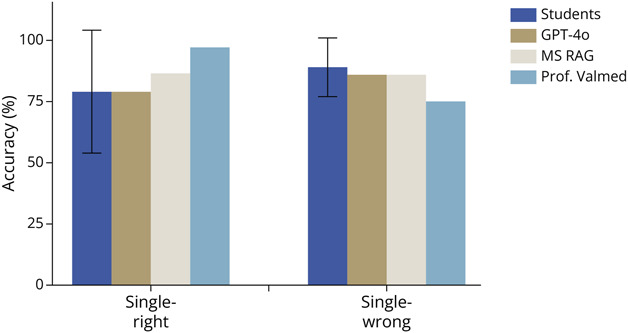
Accuracy by Question Type Accuracy achieved by MS master's students and 3 LLMs (GPT-4o, MS RAG, and Prof. Valmed) according to question type, including single-correct answer (n = 38) and single-wrong answer (n = 15) questions. Prof. Valmed demonstrated superior performance on single-correct answer questions while students performed better across single-wrong answer types, followed by GPT-4o and MS RAG. However, no significant differences were observed in χ^2^ tests. LLM = large language model; MS = multiple sclerosis; RAG = retrieval-augmented generation.

When stratified by question type, distinct performance patterns were observed among models. For single-correct answer questions (n = 38 questions), Prof. Valmed and MS RAG demonstrated better performances compared with GPT-4o, achieving accuracies of 97.1%, 86.8%, and 78.9%, respectively (Figure 2). However, differences among the models were not statistically significant (χ^2^(2) = 5.309, *p* = 0.070, Cramer *V* = 0.155). The student group performed comparably at 79.5% (SD 25.6%). For single-wrong answer questions (n = 15 questions), the students had a better accuracy (89%, SD 12.9%) while GPT-4o and MS RAG achieved similar accuracies (86.6%) and Prof. Valmed achieved 75%, overall. However, the group differences were not statistically significant (χ^2^(2) = 0.840, *p* = 0.657, Cramer *V* = 0.100).

### Performance by Difficulty Level

**Figure 3:**
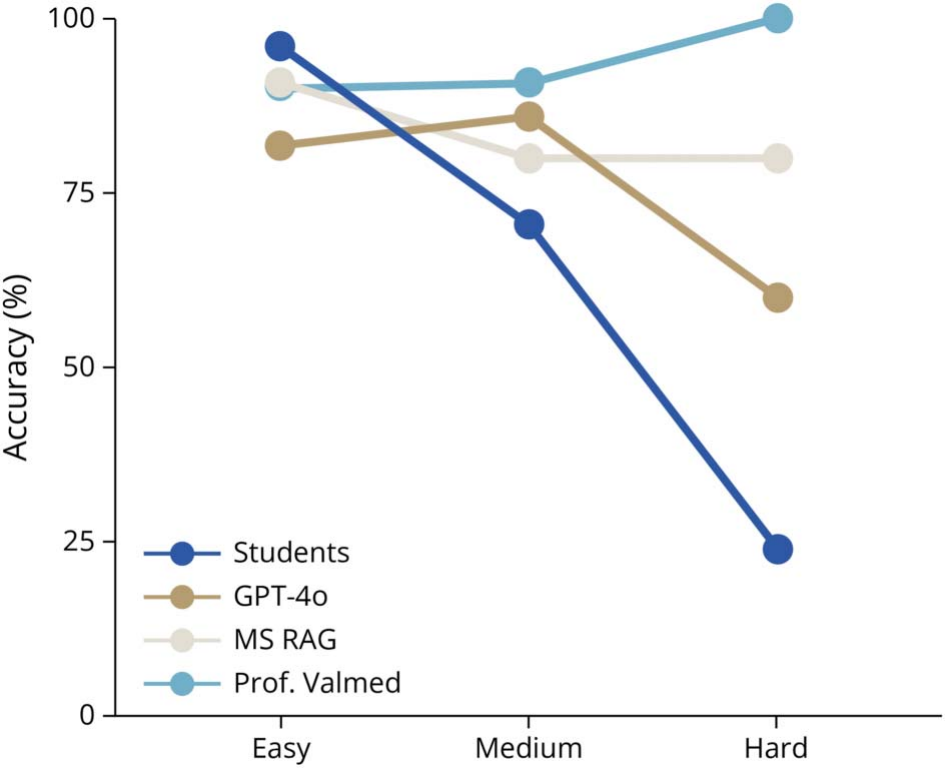
Accuracy by Question Difficulty Accuracy achieved by MS master's students and 3 LLMs (GPT-4o, MS RAG, and Prof. Valmed) according to question difficulty. Questions were stratified based on student performance: easy (>80% answered correctly), medium (50%–79%), and hard (<50%). Students outperformed LLMs on easy questions while performance on medium-difficulty questions was comparable across all groups. For hard questions, MS RAG and Prof. Valmed maintained slightly better accuracy compared with GPT-4o and the students, which experienced notable declines. However, no significant difference was observed in χ^2^ tests. LLM = large language model; MS = multiple sclerosis; RAG = retrieval-augmented generation.

Using the difficulty stratification based on student performance, the models demonstrated varying trends. For “easy” questions (students ≥80% correct, n = 33 questions), students slightly outperformed the LLMs, with 96.3% (SD 5.1%) accuracy, while GPT-4o, MS RAG, and Prof. Valmed achieved 81.8%, 90.9%, and 90.3%, respectively (Figure 3). Differences among models were not statistically significant (χ^2^(2) = 1.563, *p* = 0.458; Cramer *V* = 0.127). On “medium” questions (students 50%–79% correct, n = 15 questions), the models achieved higher accuracies than the students (70.5%, SD 8.0%), with 86.7% (GPT-4o), 80.0% (MS RAG), and 90.9% (Prof. Valmed). Again, there was no significant difference among models (χ^2^(2) = 0.637, *p* = 0.727; Cramer *V* = 0.125). For “hard” questions (students <50% correct, n = 5 questions), student accuracy dropped to 24.3% (SD 10.8%). However, GPT-4o, MS RAG, and Prof. Valmed achieved relatively better results (3/5, 4/5, and 4/4 right answers, respectively).

### Performance on Open-Ended Questions

In the subset of open-ended questions, GPT-4o produced accurate responses in 66.7% of cases, MS RAG in 76.2%, and Prof. Valmed in 85% (Table). Notably, while GPT4o and MS RAG provided 14.3% and 19.3% wrong answers, respectively, Prof. Valmed did not have any clearly wrong answer, but a higher rate of outputs lacking substantive insight into the question. The responses included statements such as, “I'm here to help with medical and health-related questions. It seems your query is outside my expertise. Please try asking a medical-related question” or “I'm sorry, I couldn't find any relevant information for your question. Please try rephrasing your question or providing more specific details to help me find better results.”

**Table:** Accuracy of Open-Ended Responses (N = 21 Questions)

	GPT4o	MS RAG	Prof. Valmed
Accurate answers, %	66.7	76.2	85
Indeterminate answers, %	19	14.3	15
Inaccurate answers, %	14.3	4.8	0

Abbreviations: MS = multiple sclerosis; RAG = retrieval-augmented generation.

“Accurate” answers fully addressed the clinical question based on predefined criteria and expert consensus. “Indeterminate” answers included safety, vague, or generic responses that did not provide sufficient information to judge clinical appropriateness. “Inaccurate” answers contained clinically incorrect or misleading information. All categorizations were independently performed by 2 MS-trained clinicians.

## Discussion

This study provides novel insights into the performance of LLMs in the context of MS-specific knowledge application in educational settings. By comparing both structured and open-ended performance, the study helps to identify key tradeoffs between general-purpose and domain-specific architectures.

GPT-4o, MS RAG, and Prof. Valmed displayed relatively similar overall accuracy rates, with a tendency toward better results for the latter 2. This suggests that differences in training data and retrieval mechanisms may influence the performance of LLMs, particularly when applied to domain-specific educational tasks. While the overall performance was comparable to postgraduate students, the differences emerged by question type and difficulty level.

LLMs demonstrated greater consistency on medium and hard questions, whereas students performed best on easier items. This may suggest that LLMs provide consistent performance as question complexity increases. However, performance differed depending on question type: single-correct answer questions yielded the highest accuracy across all groups, with Prof. Valmed showing the numerically best performance, potentially because of its alignment with structured, guideline-based queries. Conversely, students outperformed all LLMs on single-wrong answer questions, suggesting human reasoning strengths in distinguishing between near-plausible distractors.

Despite the small number of questions and statistical power, Prof. Valmed showed numerically stronger performance on medium and hard questions, suggesting strengths in structured, guideline-based reasoning. MS RAG also performed relatively well on hard questions, suggesting the value of retrieval-augmented architectures in addressing less straightforward content. These findings are consistent with previous work showing improved accuracy and relevance through dynamic retrieval mechanisms.^3,24^ RAG models could determine when additional context is needed, a critical feature for managing questions with nuances of real-world clinical decision making.^24,25^ Combining fine-tuned knowledge with selective retrieval may be essential for replicating expert-like behavior.

Although multiple-choice formats are efficient for structured evaluation, they may not fully capture the depth of reasoning and contextual navigation required in clinical practice.^26,27^ These formats primarily assess knowledge recall rather than the reasoning processes needed to handle unstructured or ambiguous scenarios. By contrast, information retrieval emphasizes identifying relevant content dynamically, reflecting the reasoning demands of real-world decision making.^28^ Retrieval performance varies depending on domain-specific fine-tuning, retrieval methods, and prompting strategies.^29^ In neurology, these are critical for ensuring that LLM-generated responses align with current medical knowledge and guidelines.

In open-ended tasks, Prof. Valmed achieved the highest accuracy, followed by MS RAG and GPT-4o. It is important to note that Prof. Valmed did not produce any inaccurate or clearly incorrect answers. Instead, in a few cases, it provided generic or limited responses, possibly because of built-in safeguards or confidence thresholds. We excluded these unanswered questions from the accuracy calculation, because penalizing abstentions would counteract the model's designed safety mechanism. Nonetheless, this methodological decision may limit comparability. Prof. Valmed's behavior may reflect an intentional safeguard mechanism prioritizing safety and guideline fidelity rather than broad contextual reasoning in probably ambiguous prompts.^3,30^ These differences emphasize the tradeoff between adaptability and cautiousness: general-purpose models may respond more flexibly but risk generating inaccurate content, whereas specialized models may prioritize reliability. This distinction is particularly relevant when considering LLM use in decision support.

Certain limitations must be acknowledged. The relatively small and fixed set of questions, although validated by MS specialists, may not capture the full complexity and variability of MS care. The limited number of items may have restricted the statistical power to detect subtle but potentially meaningful performance differences. Moreover, although multiple-choice and open-ended tasks allow standardized comparisons, they may oversimplify the complexity of clinical reasoning.^25^ The lack of an established MS-specific benchmark limits generalizability, and our inability to assess hallucination rates, explainability, or citation behavior further constrains interpretation. Leveraging structured and validated benchmarks could facilitate robust and reproducible evaluations.^31^ Finally, although most participants were neurologists or trainees, a small proportion came from related fields, introducing a minor potential confounder.

Although not evaluated in an instructional context, these results provide a basis for exploring how LLMs might be integrated into postgraduate neurology education. Beyond demonstrating accuracy comparable to postgraduate students, the models showed added usefulness on more difficult tasks, suggesting potential as complementary tools for training. Possible applications include AI tutors that provide individualized feedback on complex questions; retrieval-augmented systems that support formative assessments by aligning with evolving evidence; or supplementary review tools for learners struggling with specific reasoning tasks. These approaches could help address variability in training and ensure consistent access to evolving knowledge. It is important to note that these findings may also hold relevance for other subspecialties within neurology, where evolving guidelines and complex decision making pose similar educational challenges.

Future research should expand question sets and formats to encompass a broader spectrum of MS care. A benchmarking dataset for MS, combining multiple formats and difficulty levels, would support robust, reproducible model evaluations. Exploring the interpretability of outputs, frequency of hallucinations, and alignment with verifiable references is essential for evaluating safety and clinical utility.

Careful integration, with attention to safety, guideline adherence, and transparency, will be essential before LLMs can be considered meaningful additions to specialized neurology education. Recent innovative conceptual approaches in LLM development, such as “white listening” or reinforcement learning from human feedback, which aim to enhance transparency and controllability of outputs, underscore the growing emphasis on interpretability and safety in clinical applications.^32-34^ Incorporating such techniques into domain-specific models may further enhance reliability and trust. Moreover, analyzing the reasoning pathways behind LLM-generated answers—especially for complex items—may provide an additional pedagogical layer, enabling adaptive feedback and uncovering strengths and limitations in replicating expert-level clinical reasoning.

LLMs, particularly those with retrieval-augmented or domain-specific architectures, can apply MS-specific knowledge comparable to postgraduate students in a specialized MS curriculum. Beyond overall accuracy, the models may show added usefulness on more difficult tasks, suggesting potential as complementary tools in neurology education. However, differences in adaptability, safety constraints, and response style highlight the importance of matching LLM architecture to its intended use case. Broader validation with real-world scenarios and standardized benchmark datasets will be essential to confirm educational and clinical utility and ensure safe integration into postgraduate training.

## Glossary

AI: artificial intelligence
DIU: Dresden International University
IQR: interquartile range
LLM: large language model
MS: multiple sclerosis
RAG: retrieval-augmented generation
